# Conversion of rod-shaped gold nanoparticles to spherical forms and their effect on biodistribution in tumor-bearing mice

**DOI:** 10.1186/1556-276X-7-565

**Published:** 2012-10-11

**Authors:** Yasuyuki Akiyama, Takeshi Mori, Yoshiki Katayama, Takuro Niidome

**Affiliations:** 1 Department of Applied Chemistry, Faculty of Engineering, Kyushu University, 744 Motooka, Nishi-ku, Fukuoka, 819-0395, Japan; 2 Center for Future Chemistry, Kyushu University, 744 Motooka, Nishi-ku, Fukuoka, 819-0395, Japan; 3 International Research Center for Molecular Systems, Kyushu University, 744 Motooka, Nishi-ku, Fukuoka, 819-0395, Japan

**Keywords:** Aspect ratio, Gold nanoparticles, Gold nanorods, Laser, Biodistribution

## Abstract

Gold nanorods that have an absorption band in the near-infrared region and a photothermal effect have been used as nanodevices for near-infrared imaging and thermal therapy. Choice of the optimal shape of gold nanorods which relates optical properties and *in vivo* biodistribution is important for their applications. In the present study, to investigate the relationship between the shape of gold nanorods and their biodistribution after intravenous injection, we first prepared two types of gold nanorods that had distinct aspect ratios but had the same volume, zeta potential, and PEG density on the gold surface. Biodistributions of the two types of gold nanorods after intravenous injection into tumor-bearing mice were then compared. Although a slight difference in accumulation in the spleen was observed, no significant difference was observed in the liver, lung, kidney, and tumors. These results suggest that biodistribution of the gold nanorods in the aspect ratio range of 1.7 to 5.0, diameter of 10 to 50 nm, and volume of approximately 4 × 10^3^ nm^3^ was dependent mainly on surface characteristics, PEG density, and zeta potential.

## Background

Gold nanorods are tiny, rod-shaped gold particles. They have two distinctive different absorption bands derived from the transverse and longitudinal surface plasmon resonance of free electrons in the visible and near-infrared (NIR) regions, respectively [1,2]. Gold nanorods absorb NIR light, which is suitable for *in vivo* applications such as imaging and for photoradiation therapy because of maximal penetration of light into tissues [3]. Gold nanorods have a photothermal effect, i.e., the adsorbed light energy is converted to heat [4]. Therefore, gold nanorods are expected to be contrast agents for NIR imaging and exothermic nanodevices for photothermal therapy.

Gold nanorods are prepared in the presence of the cationic detergent 1-hexadecyltrimethylammonium chloride (cetyltrimethylammonium chloride; CTAB), which acts as a stabilizer of gold nanorods. To apply gold nanorods as medical nanodevices, biocompatible gold nanorods have been prepared by coating with phosphatidylcholine [5] or by modifying gold nanorods with polyethylene glycol (PEG) [6,7]. PEG-modified gold nanorods show high dispersion stability, high circulation stability in the blood after intravenous injection into mice [7], and accumulation in tumors mediated by the enhanced permeability and retention (EPR) effect [8,9]. The PEG-modified gold nanorods have been applied to NIR imaging and photodynamic/photothermal therapy of tumors [8,10-13].

The size and aspect ratio of gold nanorods are important factors of their uptake into cells [14-16] and biodistribution [17-20]. Arnida et al. reported that rod-shaped gold nanoparticles accumulated more efficiently in tumors and less efficiently in the liver compared with sphere-shaped gold nanoparticles [20]. However, the compared gold nanorods and gold nanospheres had different volumes and zeta potentials that affected cellular uptake (e.g., phagocytosis) [21]. Therefore, if we examine the effect of the geometry of gold nanoparticles on biodistribution, other parameters should be equivalent.

In the present study, we prepared two types of gold nanorods that had distinct aspect ratios but had an identical volume, zeta potential, and surface structure. Biodistributions of these nanorods after intravenous injection into tumor-bearing mice were then compared.

## Methods

### Animals

Animal experiments were undertaken according to the Guidelines for Animal Care and Use Committee, Kyushu University (Fukuoka, Japan). Male ddY mice (Kyudo Co., Ltd., Saga, Japan) aged 5 to 6 weeks were used. Mice were maintained in a temperature-controlled environment at 24°C with a 12-h light-dark cycle. They were provided with drinking water and food *ad libitum*.

### Preparation of gold nanorods of lower aspect ratio from higher ones

Gold nanorods prepared by our research team were obtained from Mitsubishi Materials Corp. (Tokyo, Japan) and Dai Nippon Toryo Co. Ltd. (Tokyo, Japan) [22]. The mean length and width of the as-obtained gold nanorods were 49.6 ± 9.7 nm and 10.6 ± 3.2 nm, respectively (aspect ratio, 5.0 ± 1.2). A suspension of gold nanorods containing CTAB was stored at a constant temperature (37°C). Gold nanorods were modified with PEG chains. Briefly, the gold nanorods in the suspension were centrifuged at 14,000 × *g* for 10 min at room temperature, decanted, and re-suspended in water to remove excess CTAB. Thiol-terminated mPEG solution (molecular weight approximately 5,000 Da; NOF Corporation, Tokyo, Japan) was added to the gold nanorod suspension at a PEG/gold molar ratio of 1.0. The mixture of PEG solution and gold nanorod suspension at a final gold concentration of 1 mM was stirred for 24 h at room temperature. NIR pulsed laser light (Nd:YAG 1064 nm, 750 mW, 20 Hz, approximately 6 mm in beam diameter) irradiated the mixture for 5 min. The mean length and width of gold nanorods irradiated with the NIR pulsed laser light were 27.0 ± 8.2 nm and 17.2 ± 3.7 nm, respectively (aspect ratio, 1.7 ± 0.9). A control mixture was not irradiated with the NIR pulsed laser light. These mixtures were stirred for another 24 h at room temperature and centrifuged twice at 14,000 × *g* for 10 min at room temperature, decanted, and re-suspended in water to remove remaining CTAB and excess PEG reagent.

### Characterization of PEG-modified gold nanorods

The absorption spectra of gold nanorods in the visible-to-NIR light regions were measured with a V-670 spectrophotometer (Jasco, Tokyo, Japan). Gold nanorods and the PEG layer on the gold nanorods were imaged with a transmission electron microscope (JEM-2010, JEOL, Tokyo, Japan) after staining the PEG layer with 1% phosphotungstic acid [23]. Particle size distribution, surface area, and volume were calculated from transmission electron microscopy (TEM) images of 100 particles. Amounts of PEG modified on the gold nanorods were evaluated by elemental analyses according to our previous publication [24]. Zeta potentials of gold nanorod suspensions in 0.2 M sodium phosphate buffer (pH 7.4) were evaluated using a Malvern Zetasizer Nano ZS (Malvern, Worcestershire, UK).

### Biodistribution of gold nanorods

Tumor-bearing mice were used in biodistribution experiments and were prepared as follows: Colon-26 cells: mouse colon carcinoma cells were cultured in RPMI 1640 medium containing 10% fetal bovine serum, 100 U/mL penicillin, 100 μg/mL streptomycin, and 0.25 μg/mL amphotericin B. Cells were incubated at 37°C under a 5% CO_2_-humidified atmosphere. Incubated colon-26 cells were injected subcutaneously into the abdomen at 2 × 10^6^ cells in 100 μL of Hank's balanced salt solution per mouse (ddY, male, 5 weeks, 29 to 35 g). They were allowed to grow for 5 days, wherein the tumors reached approximately 5 to 7 mm in diameter. PEG-modified gold nanorods were injected into tumor-bearing mice via the tail vein. Doses of PEG-modified gold nanorods with an aspect ratio of 5.0 and PEG-modified gold nanorods with an aspect ratio of 1.7 were at 1.01 and 1.05 mM of gold concentration in 10 μL/g body weight of 5% glucose solution, respectively. Mice were sacrificed 72 h after injection. Several hundred microliters of blood were collected from the heart and immediately mixed with 1 mg ethylenediamine tetra-acetic acid. The liver, lung, spleen, and kidney, as well as tumors, were collected. These samples were added to approximately 4 mL aqua regia and then heated overnight at 90°C to 100°C. Samples were diluted in 0.5 M HCl to the appropriate volume. Gold concentrations were quantified by inductively coupled plasma mass spectrometry (ICP-MS) using an Agilent 7500c system (Agilent. Tokyo, Japan). ICP-MS measurement was done with thallium as the internal standard.

## Results and discussion

### Preparation of PEG-modified gold nanorods with distinct aspect ratios

**Figure 1:**
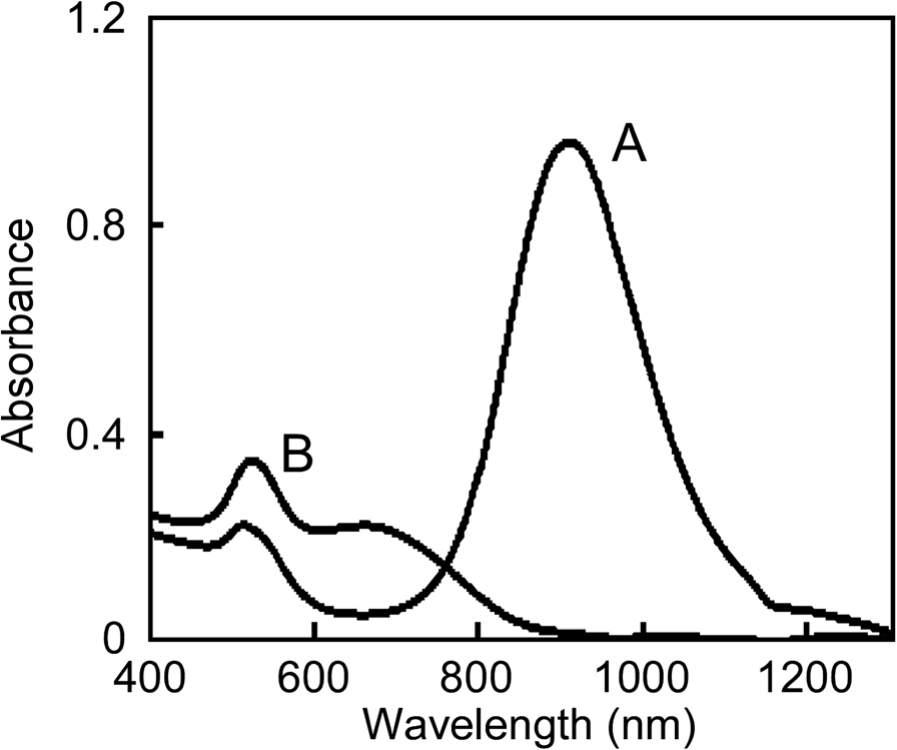
Absorption spectra of PEG-modified gold nanorods treated without (A) and with (B) near-infrared pulsed laser light.

**Figure 2:**
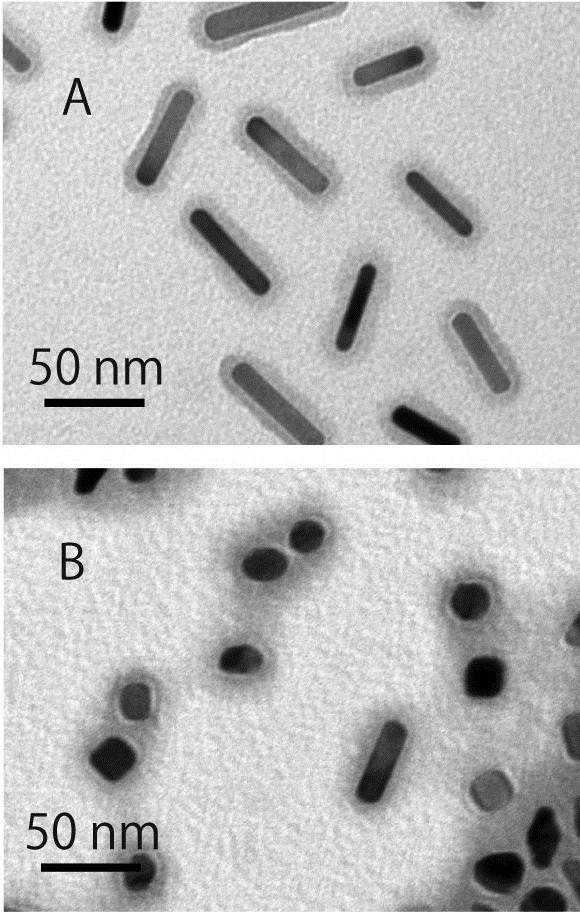
** TEM images of PEG-modified gold nanorods treated without (A) and with (B) near-infrared pulsed laser light.** Samples were stained with 1% phosphotungstic acid; thereafter, images were taken. Scale bars = 50 nm.

**Figure 3:**
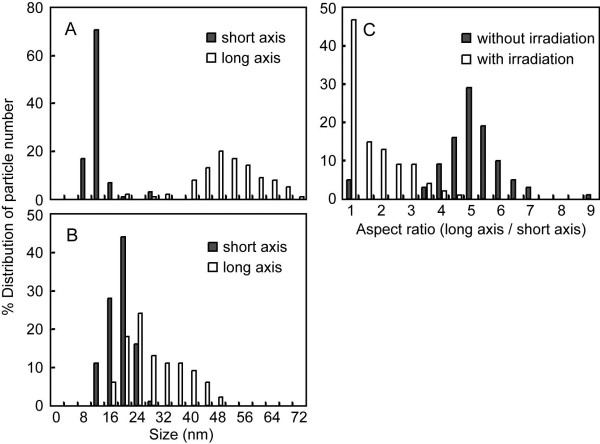
** Size distribution and aspect ratios of PEG-modified gold nanorods.** Size distribution of PEG-modified gold nanorods treated without (**A**) and with (**B**) near-infrared pulsed laser light. Closed and open bars show the size distributions of the short and long axes of gold nanorods, respectively. (**C**) Aspect ratios of PEG-modified gold nanorods without (closed bars) and with (open bars) pulsed laser light.

To clarify the effect of the aspect ratio of gold nanorods on biodistribution in mice, we prepared PEG-modified gold nanorods with distinct aspect ratios. Gold nanorods can be melted and reshaped by irradiation with NIR pulsed laser light at the absorption wavelength of the gold nanorods [24-26]. Based on those reports, the original gold nanorods with higher aspect ratio were transformed to those of lower aspect ratio by irradiation with laser light. After irradiation, maximum absorption and the wavelength at the maximum absorption of the gold nanorods in the NIR region decreased and shifted towards a shorter wavelength (Figure 1). TEM images revealed that the irradiated gold nanorods showed mainly spherical or elliptical shapes and partly rod shapes (Figure 2). For gold nanorods with and without irradiation, PEG modified on the gold nanorods was confirmed from a gray layer of PEG stained with phosphotungstic acid. Particle sizes of gold nanorods in the TEM images were measured; then, their distributions were plotted (Figures 3A, B). Laser irradiation made the size of the short axis of the gold nanorod increase and the size of the long axis decrease. As a result, the size distribution of the short and long axes overlapped. Aspect ratios were calculated and then plotted (Figure 3C). Half of the irradiated gold nanorods were transformed to a spherical shape (aspect ratio, 1.0). The other irradiated gold nanorods were transformed to an elliptical shape. 

Several characteristics of the gold nanorods are summarized in Table 1. The particle length of the short axis and long axis increased from 10.6 to 17.2 nm and decreased from 49.6 to 27.0 nm by laser irradiation, respectively. Laser irradiation made the aspect ratio decrease from 5.0 to 1.7. The surface area and volume of the gold nanorods were calculated on the assumption that a nanoparticle with an aspect ratio of 1.0 was a sphere and a nanorod with an aspect ratio >1.0 was a cylinder with a hemisphere domain at both ends. The surface area decreased from 1.61 × 10^3^ nm^2^ to 1.42 × 10^3^ nm^2^ by laser irradiation. The volume did not change significantly after irradiation. Previously, it was reported that irradiation of gold nanorods by pulsed laser light caused fragmentation into smaller particles [26] or fusion into larger particles [27,28]. However, in the irradiation condition described here, neither fragmentation nor fusion of the gold nanorods was observed in TEM images. This was probably because the intensity of the pulsed laser light was not sufficient to induce fragmentation of the gold nanorods, and the PEG layer on the gold nanorods disturbed the fusion between gold nanorods. 

**Table 1:** Characteristics of PEG-modified gold nanorods treated without and with near-infrared pulsed laser light

	**Without irradiation**	**With irradiation**
Particle size of short axis (nm)	10.6 ± 3.2	17.2 ± 3.7
Particle size of long axis (nm)	49.6 ± 9.7	27.0 ± 8.2
Aspect ratio	5.0 ± 1.2	1.7 ± 0.9
Surface area (×10^−3^ nm^2^)	1.61 ± 0.43	1.42 ± 0.40
Volume (×10^−3^ nm^3^)	4.02 ± 1.81	4.32 ± 1.70^a^
Number of modified PEG on one particle (×10^−2^)	9.50 ± 4.28	8.25 ± 3.25
Area occupied by one PEG molecule (nm^2^)	1.82 ± 0.29	1.82 ± 0.33^a^
Zeta potential (mV)	0.48 ± 1.66	1.83 ± 3.34^a^

Data are the mean ± standard deviation. ^a^No significant difference from the value of gold nanorods without laser irradiation.

The amount of modified PEG on gold nanorods was measured by elemental analyses (Table 1) [24]. The number of modified PEG molecules on one particle decreased from 9.50 × 10^2^ to 8.25 × 10^2^ by laser irradiation. The actual release of PEG from the gold surface to supernatant has been already confirmed at a similar condition in our previous report [24]. However, the area occupied by one PEG molecule as PEG density on the gold nanorods and the zeta potentials of the gold nanorods did not change after laser irradiation. These data suggested that the laser irradiation of gold nanorods resulted in transformation from a rod shape to a spherical shape without changing the volume, PEG density, or zeta potential.

### Biodistribution in tumor-bearing mice

**Figure 4:**
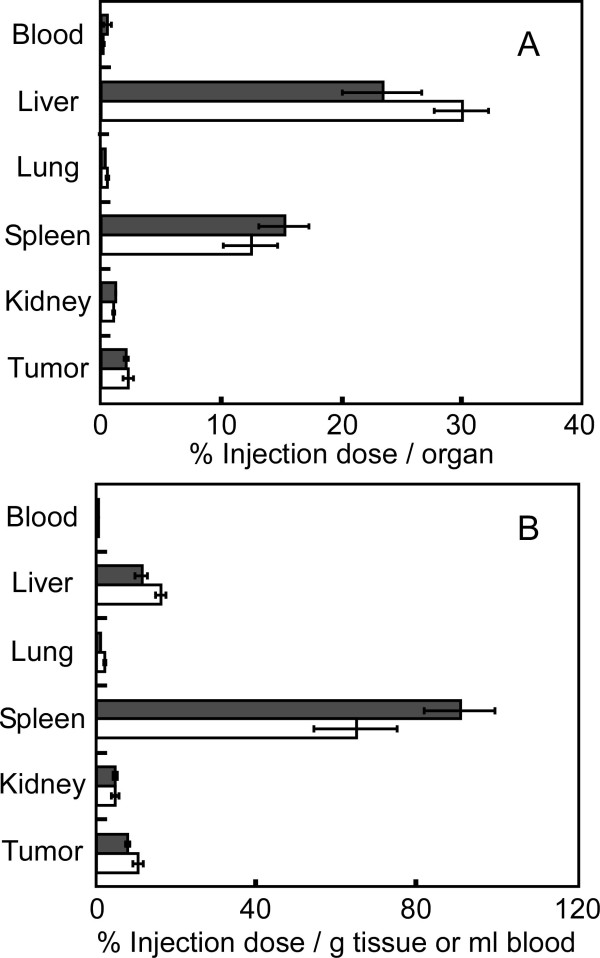
** Biodistribution of PEG-modified gold nanorods in tumor-bearing mice.** The biodistribution of PEG-modified gold nanorods at 72 h after intravenous injection was compared. Closed bars and open bars indicate the biodistribution of PEG-modified gold nanorods at aspect ratios of 5.0 (*n* = 5) and 1.7 (*n* = 4), respectively. (**A**) The percentage of injected dose per organ and (**B**) percentage of injected dose per gram of tissue or milliliter of blood, respectively. Bars are the standard error from the mean.

We compared the biodistribution of PEG-modified gold nanorods with distinct aspect ratios but with the same volume, PEG density, and zeta potentials. Prepared gold nanorods with aspect ratios of 5.0 and 1.7 were injected intravenously into tumor-bearing mice to which colon-26 cells were subcutaneously injected. Mice were sacrificed 72 h after the injection, and the collected organs were dissolved using aqua regia. At this time point, it has been already shown that little amount of PEG-modified gold nanorods was observed in the blood and that most gold nanorods were distributed to organs [7,9]. Gold concentrations in the samples were measured by ICP-MS. Both types of injected gold nanorods accumulated mainly in the liver and spleen (Figure 4A). Most gold nanorods with aspect ratios of 5.0 and 1.7 were probably cleared by the reticuloendothelial system (RES) in the liver and spleen [28]. 

Figure 4B shows the concentration of accumulated gold nanorods in the organs as percentage of injected dose per gram of tissue or milliliter of blood. The concentration is an important parameter for photothermal therapy and bioimaging mediated by gold nanorods. Accumulation of gold nanorods with an aspect ratio of 1.7 in the spleen was slightly lower than that of gold nanorods with an aspect ratio of 5.0, although statistical significance was low (0.1 > *p* > 0.05; Figure 4B). In the case of the liver, lung, and kidney, no difference between the gold nanorods with aspect ratios of 1.7 and 5.0 was observed. No difference was observed in the tumors, suggesting that a change in the shape of the gold nanorods was not sufficient to influence the EPR effect in the tumors. The slight difference observed in the spleen may have been due to physical filtration in this organ as observed for larger particles (>200 nm) [29,30]. It may not have been due to changes in the contribution of the RES in the spleen because a significant difference in the accumulation in the liver (where many Kupffer cells (macrophage-like cells) constitute the RES at sinusoids) was not observed. These results suggested that the shape of the gold nanorods barely affected the biodistribution of gold nanorods after intravenous injection into tumor-bearing mice except for that in the spleen (though the change in biodistribution was slight). Previously, we compared biodistributions of several types of gold nanorods, which have different zeta-potentials and PEG densities on the gold surface but have the same aspect ratio [8,9]. In those studies, the surface structure of the gold nanorods strongly affected their biodistributions after intravenous injection. Therefore, it can be concluded that the biodistribution would be more affected primarily by the surface characteristics of the gold nanorods than the aspect ratio.

## Conclusions

In the present study, the shape of the original gold nanorods was changed by irradiation with NIR light to a spherical form. Then, we obtained two types of PEG-modified gold nanorods with distinct aspect ratios but with the same volume, PEG density, and zeta potential. The change in the aspect ratio of the gold nanorods did not affect the biodistribution appreciably. These results suggest that the biodistribution of gold nanorods in the aspect ratio range of 1.7 to 5.0 and size of 10 to 50 nm at a volume of approximately 4 × 10^3^ nm^3^ is dependent mainly on the surface characteristics, PEG density, and zeta potential. If the application of gold nanorods to therapeutic use is considered, the gold nanorods circulating in the blood or other tissues such as tumors are irradiated with NIR light. Irradiation with the pulsed laser light will induce the shape change of the gold nanorods and release of molecules bound on the gold nanorods without changing their biodistribution. On the other hand, irradiation with continuous wave laser light can heat the gold nanorods continuously without changing the shape of the gold nanorods [24]. Thus, combinations of gold nanorods and different modes of light irradiation provide us various drug delivery/release and heating systems, and it will be an advantage of gold nanorods over other types of nanoparticles. The results obtained in this study will be used as basic information on the *in vivo* application not only of gold nanorods with various aspect ratios, but also of other types of anisotropic nanoparticles.

## Abbreviations

CTAB: cetyltrimethylammonium chloride 1-hexadecyltrimethylammonium chloride; EPR: enhanced permeability and retention; ICP-MS: inductively coupled plasma mass spectrometry; NIR: near infrared; PEG: polyethylene glycol.

## Competing interests

The authors declare that they have no competing interests.

## Authors’ contributions

YA designed and carried out all the experiments and drafted the manuscript. MT and KY supported the experiments. TN provided the idea, supervised the study, and wrote the manuscript. All authors read and approved the final manuscript.
